# Restoring the complexity of walking in the elderly and its impact on clinical measures around the risk of falls

**DOI:** 10.3389/fnetp.2025.1532700

**Published:** 2025-04-02

**Authors:** Samar Ezzina, Simon Pla, Didier Delignières

**Affiliations:** ^1^ Union Nationale Sportive Léo Lagrange, Paris, France; ^2^ Euromov DHM, University of Montpellier, Montpellier, France

**Keywords:** rehabilitation, aging, locomotion, complexity, falls, network physiology

## Abstract

**Introduction:** The hypothesis of the loss of complexity with aging and disease has received strong attention. Especially, the decrease of complexity of stride interval series in older people, during walking, was shown to correlate with falling propensity. However, recent experiments showed that a restoration of walking complexity in older people could occur through the prolonged experience of synchronized walking with a younger companion. This result was interpreted as the consequence of a complexity matching effect.

**Experiment:** The aim of the present study was to analyze the link between the restoration of walking complexity in older people and clinical measures usually used in the context of rehabilitation or follow-up of older people.

**Results:** We evidenced a link between restoring complexity, improving overall health and reducing fear of falling. In addition, we showed that 3 weeks of complexity matching training can have a positive effect on complexity up to 2 months post-protocol. Finally, we showed that the restoration of walking complexity obtained in the previous works is not guide-dependent.

## 1 Introduction

Walking is regulated through spinal and supraspinal networks allowing to adapt to intrinsic and extrinsic perturbations (Ravi et al., 2020). The efficiency of regulation is enabled by the complexity of these underlying networks, that is, by the richness of the interactions between their multiple components and subsystems. The more complex the underlying networks, the more stable and adaptable locomotion is. The complexity of networks can be revealed by the temporal structure of the time series produced during locomotion, and in particular by the series of durations of successive steps. Complex systems produce series characterized by long-range correlations, meaning that one-stride interval is correlated to stride intervals at relatively distant time points. In contrast, simpler systems, unable to achieved resistance to perturbations, result in more random series.

Several studies have supported the hypothesis of a loss of complexity linked to age or pathologies (Lipsitz and Goldberger, 1992; Goldberger et al., 2002; Harrison and Stergiou, 2015; Stergiou and Decker, 2011). Especially, Hausdorff et al. (1997) showed that the decrease in complexity in the dynamics of stride duration in older people was correlated with the propensity to fall (see also Herman et al., 2005; Hausdorff, 2007; Kurz et al., 2010; Gonabadi et al., 2021). Two recent studies explored the hypothesis of the restoration of walking complexity in older people (Almurad et al., 2018; Ezzina et al., 2021). These experiments showed that it was possible to restore walking complexity through a prolonged training in arm-in-arm walking. These results were interpreted through the concept of complexity matching, introduced by West et al. (2008), and suggesting a harmonization of complexities when two complex systems interact (Abney et al., 2014; Delignières and Marmelat, 2014; Coey et al., 2016; Almurad et al., 2017). A recent study by Ezzina et al. (2021) showed that synchronized arm-in-arm walking is dominated by a complexity matching effect, characterized by a close harmonization of the complexities of the two partners. This work shows that the level of complexity developed during arm-in-arm walking by older participants was close to that exhibited by the young partner during solo trials, which means that the most complex system attracts the less complex one. These results are consistent with the hypothesis of Mahmoodi et al. (2018), Mahmoodi et al. (2020) considering that when two systems with different complexity levels interact, the least complex system is “attracted” by the most complex, yielding an increase of the complexity of the former. Moreover, the study of Ezzina et al. (2021) showed that 3 weeks of regular complexity matching training were enough to restore complexity in older people, and this restoration was persistent up to 6 weeks post-protocol.

However, these previous experiments present some limitations. First, the number of young partners involved in the rehabilitation protocol remained low, one in Almurad et al. (2018), two in Ezzina et al. (2021), and a possible experimenter effect could be advocated for explaining the obtained results. Second, the structures of care and follow-up of older people need arguments on the link between the restoration of the complexity of walking in older patients and the reduction of the propensity to fall. Fall is not an incident due to advancing age but the consequence of an accumulation of several risk factors, combined with a weakened general condition (Rubenstein, 2006; Montero-Odasso et al., 2022). Therefore, there are several risk factors for falls, and it is possible to assess them. So, we have spotted some tests, commonly used in the follow-up of older people and recommended by health authorities, for the assessment of the risk of falls.

Our hypotheses were as follows:

- If an older person is invited to walk in synchrony, arm-in-arm with a healthy young guide, we should observe a complexity matching effect within the dyad.
- Given the asymmetry of complexities (with older participants having lower levels of complexity than their guides), the complexity matching effect should lead to an increase in complexity in the older participants.
- Prolonged synchronous walking training with healthy partners should induce a lasting restoration of complexity in older participants.
- Restoring complexity should lead to better performances on global clinical tests to assess the risk of falls.

## 2 Methods

### 2.1 Participants

36 participants (14 male and 22 female, mean age: 75.4 yrs, *SD (Standard deviation) =* 6.2) were involved in the experiment. They were recruited within associations or through an advertisement in a local newspaper. They presented no contraindication to the practice of autonomous walking (respiratory, cardiovascular, musculoskeletal, or neurological pathologies). They were randomly assigned to two groups, experimental group (9 male and 11 female, mean age: 77 yrs, *SD =* 6.7, mean weight: 66.6 kg, *SD =* 10.8, mean height: 165.1 cm, *SD =* 9.3), and control group (5 male and 11 female, mean age: 73.5 yrs, *SD =* 5.1, mean weight: 68.6 kg, *SD =* 14.8, mean height: 163.1 cm, *SD =* 8.1).

Due to logistical constraints and the complexity of implementing the experiment, participants were randomly assigned to either the control or experimental group based on the order of their registration in our volunteer listing. However, their allocation to **Guide 1 or Guide 2** depended on the planning of the guides, ensuring another level of randomization.

The inclusion criteria for our study specified that participants had to be aged 65 years or older, with no contraindications to physical activity. They also needed to have no pathological gait disorders and to be able to walk independently without assistance. All participants were residents of Montpellier or its surrounding areas.

Two young volunteers in civic service were recruited as partners (a woman and a man, aged 20 and 21, respectively). Each guide walked with 10 participants from the experimental group.

### 2.2 Experimental procedure

This study was carried out under the same conditions as our previous study (Ezzina et al., 2021), namely, on an indoor athletics track (circumference 200 m). Participants were asked to perform gait training for three consecutive weeks. Each week consisted of three training sessions, performed on Monday, Wednesday and Friday. During each session, participants were instructed to complete four 15-min walking sequences. During the Monday session, participants began with a solo sequence during which they had to walk alone, in the most regular way, for 15 min, at their preferential speed. This solo sequence allowed us to assess the complexity of the series of step lengths produced by the participants at the beginning of each week.

**FIGURE 1:**
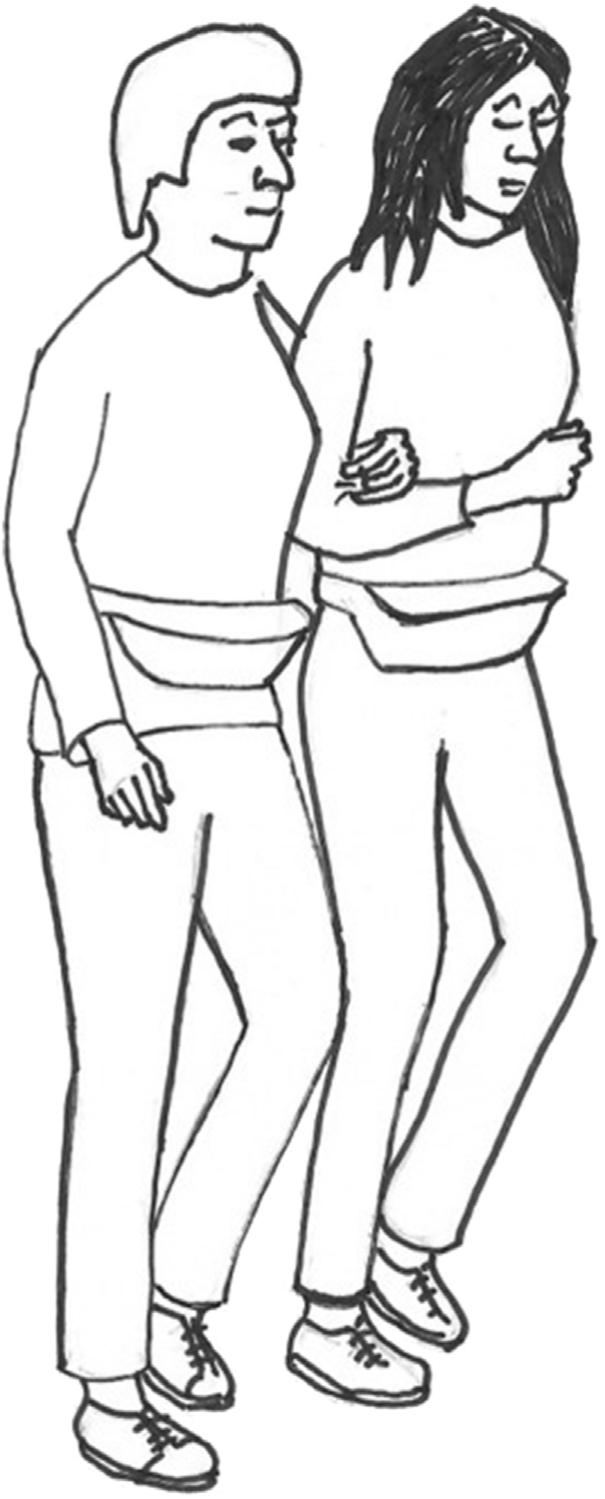
Illustration of the arm-in-arm walking condition. The participants are explicitly instructed to synchronize their steps with those of their guide.

In the experimental group, each participant was accompanied by his guide for all the other trials of the walking sequences of the week (three sequences on Monday, four sequences on Wednesday and four sequences on Friday). Participants walked arm-in-arm with their guide (Figure 1). The two guides received specific instructions for maintaining a close arm-in-arm coupling with each participant, during the whole trials. Previous experiments have shown that arm-in arm walking spontaneously elicited a complexity matching effect. Guides were also asked to adapt their walking speed with that of each senior participant. Participants were instructed to synchronize their steps with those of their guide.

For the control group, we formed pairs of participants. The duo was invited to walk together, side-by-side but without physical contact and without any synchronization instructions. In this control group, the guides supervised the sessions and did not walk with the participants. Between two successive walking sequences, the participants had a rest of about 10–15 min. All participants, in both groups, performed the same amount of training (36 sequences, 9 h of walking). The difference between the experimental group and the control group was the presence of a young guide, physical contact and synchronization of steps while walking.

Participants also performed a solo test, called post-protocol test, 4 days after the end of the training period (during the second session of clinical tests, see below).

Finally, the participants performed two post-tests (solo sequence) at one and 2 months after the end of the walking protocol. Due to the COVID-19 pandemic, the experiment was interrupted, and eight participants were unable to complete the post-tests: four from the experimental group and four from the control group. This forced us to adapt statistical tests.

This study was conducted in accordance with the 1964 Helsinki Declaration and validated by the Euromov International Review Board (No. 1711C). Participants signed an informed consent form and were not rewarded for their participation. All the data collected as part of this study were published on the open data platform: https://doi.org/10.57745/JCUYLT.

### 2.3 Clinical tests

A battery of clinical tests, used in the geriatrics department of the Montpellier University Hospital, was proposed to participants, 4 days before and 4 days after the rehabilitation protocol. The order of the tests in this battery was randomized by a computer for each participant to eliminate any order effect. These tests were carried out within an experiment box.

The test battery included the short Physical Performance Battery (SPPB Guralnik et al., 1995; Volpato et al., 2011; Veronese et al., 2014), the Timed Up and Go test (Podsiadlo and Richardson, 1991; Shumway-Cook et al., 2000; Schoene et al., 2013), a test of unipodal balance (Barnett et al., 2003), a hand grip strength test (Bohannon, 2008), the International Falls Efficacy Scale (Kempen et al., 2007; Young and Williams, 2015) and the 6-min walking test (6MWTButland et al., 1982; Bautmans et al., 2004).

### 2.4 Data collection

Stride series were collected using the same experimental device as in the previous experiment: soles containing force-sensitive sensors in the shoes, the participants wore a bag on the belt, containing the box used to collect data from real-time sensors (see Ezzina et al., 2021).

Since the control group did not have a duet streak, we only recorded their solo walking streaks earlier in the week. For the hand grip strength test, we used the KForce Grip tool to measure the force of participants. The KForce Grip has an acquisition frequency of 75 Hz, an accuracy of 100 g and weights 200 g.

### 2.5 Data analyses

In this article, all our analyzes were carried out on the series of stride times for the right leg. Each raw series contained between 700 and 1300 data points. When analyzing the data from this experiment, we noted local trends related to the periods of increasing or decreasing walking speed, especially at the beginning of the series, due to the time it took for participants to achieve a comfortable speed. Therefore, as we know that fractal analyzes can be skewed due to local trends in the series, these acceleration/deceleration phases were removed of each series.

The series subjected to the treatment had for the solo sequences an average length of 624 points (*SD* = 93.3, max = 1197.8, min = 470) and for the duos sequences an average of 660 points (*SD* = 70.9, max = 815.4, min = 460). Most of the recorded series presented the minimum number of points required for a valid fractal analysis (Delignières et al., 2006).

We used the analysis of detrended fluctuations (DFA, Peng et al., 1994) to estimate the complexity of each data series. The DFA method works as follow. The *x*(*t*) series is first integrated, by computing for each *t* the accumulated departure from the mean of the whole series (Equation 1):

$$X\left(k\right)=\sum_{i=1}^{k}\left[x\left(i\right)-\bar{x}\right]$$ (1)

This integrated series is then divided into non-overlapping intervals of length *n*. In each interval, a least squares line is fit to the data. The series *X(t)* is then locally detrended by substracting the theoretical values *X*_*n*_(*t*) given by the regression. For a given interval length *n*, the characteristic size of fluctuation for this integrated and detrended series is calculated by (Equation 2).

$$F=\sqrt{\frac{1}{N}\sum_{k=1}^{N}{\left[X\left(k\right)-X_{n}\left(k\right)\right]}^{2}}$$ (2)

This computation is repeated over all possible interval lengths (in the present work, the shortest length was 10, and the largest *N*/2). *F* increases with interval length *n*. A power law is expected, as Equation 3

F∝nα (3)

*α* is expressed as the slope of a double logarithmic plot of *F* as a function of *n. α* = 0 corresponds to an uncorrelated series (white noise), and *α = 1* to a long-range correlated series (1/*f* noise).

We used the algorithm proposed by Almurad and Delignières (2016), which was found to result in a better estimate of the scaling exponent and is recommended by Ravi et al. (2020) in their guidelines on DFA. It should be noted that regarding the clinical objective of this study, Gonabadi et al. (2021) showed that DFA was able to differentiate adult fallers and non-fallers. We limited our statistical approach to this monofractal analysis, which seems sufficient for the analysis of gait complexity (Ivanov et al., 2009).

### 2.6 Statistical analyses

In order to assess the effects of the experimental protocol on the complexity of the series of steps in the solo trials, we applied a 3-factor ANOVA: 2 (Group) X 4 (Test) X 2 (Guide), with repeated measures on the second factor (including the three solo tests of the training protocol and the post-protocol test). All 36 participants were included in this first analysis. We used the *post hoc* Fisher’s Least Significant Difference (LSD) test to locate significant effects.

To account for the two post-tests (one and 2 months after the training protocol), we performed a second 2-factor ANOVA: 2 (Group) X 6 (Test), with repeated measures on the second factor (including the three solo tests of the training protocol, the post-protocol test, and the two post-tests). Only 22 participants were included in this analysis.

We applied the windowed detrended cross-correlation analysis (WDCC) proposed by Roume et al. (2018) to assess the nature and strength of synchronization within dyads during pair trials. The WDCC calculates the cross-correlation function in short 15-point windows, with offsets ranging from −10 to 10. The data is linearly detrended in each interval before calculating the cross-correlation coefficients. A sliding window procedure is used to obtain multiple evaluations of the cross-correlation function, and finally an average function is calculated. The significance of the cross-correlation coefficients was checked with two-tailed localization tests, comparing the obtained values to zero (Roume et al., 2018).

In order to assess the effect of the training protocol on clinical measures, we applied a 2-factor ANOVA for each test: 2 (Group) x 2 (Performance), with repeated measurement on the second factor (performance achieved before and after the walking protocol).

## 3 Results

### 3.1 Restoration of complexity

**FIGURE 2:**
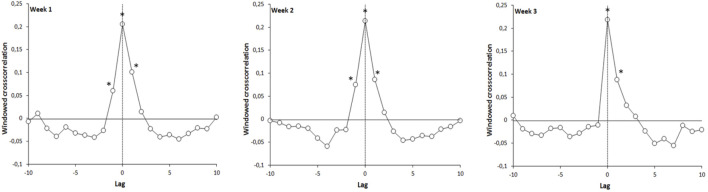
Evolution of the windowed detrended cross-correlation function, for the experimental group, during the 3 weeks of the experiment: coefficient significantly different from zero (p < 0.05).

**FIGURE 3:**
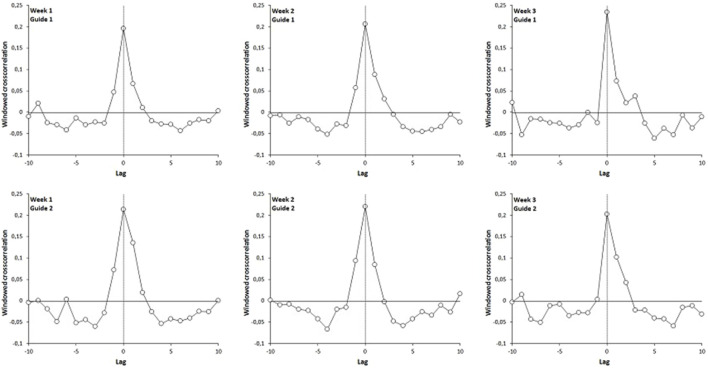
Evolution of the windowed detrended cross-correlation function, for the experimental group, during the 3 weeks of the experiment. Top figures: guide group 1, bottom figures: guide group 2.

This study confirmed that synchronized arm-in-arm walking was dominated by a complexity matching effect. In Figure 2, we present the mean WDCC functions for the 3 weeks of the protocol, for the experimental group (the control group not having been accompanied by a guide). These functions systematically exhibited a positive peak at lag 0, revealing an immediate complexity matching effect (from the first week), due to synchronization within the dyad. WDCC functions also revealed significant positive peaks at lag −1 and lag 1, suggesting a discrete correction process for asynchronies (see Roume et al., 2018) for a more detailed analysis of the properties of the WDCC). This discrete process was asymmetric, suggesting that participants corrected current stride duration based on previous asynchrony with their guide. In addition, we notice in Figure 3, presenting the average WDCC functions during the 3 weeks of protocol for guide 1 (top figures) and guide 2 (bottom figures), that the functions obtained with the 2 guides are quite similar. These results confirm that the complexities matching effect is systematically present regardless of the guide.

**FIGURE 4:**
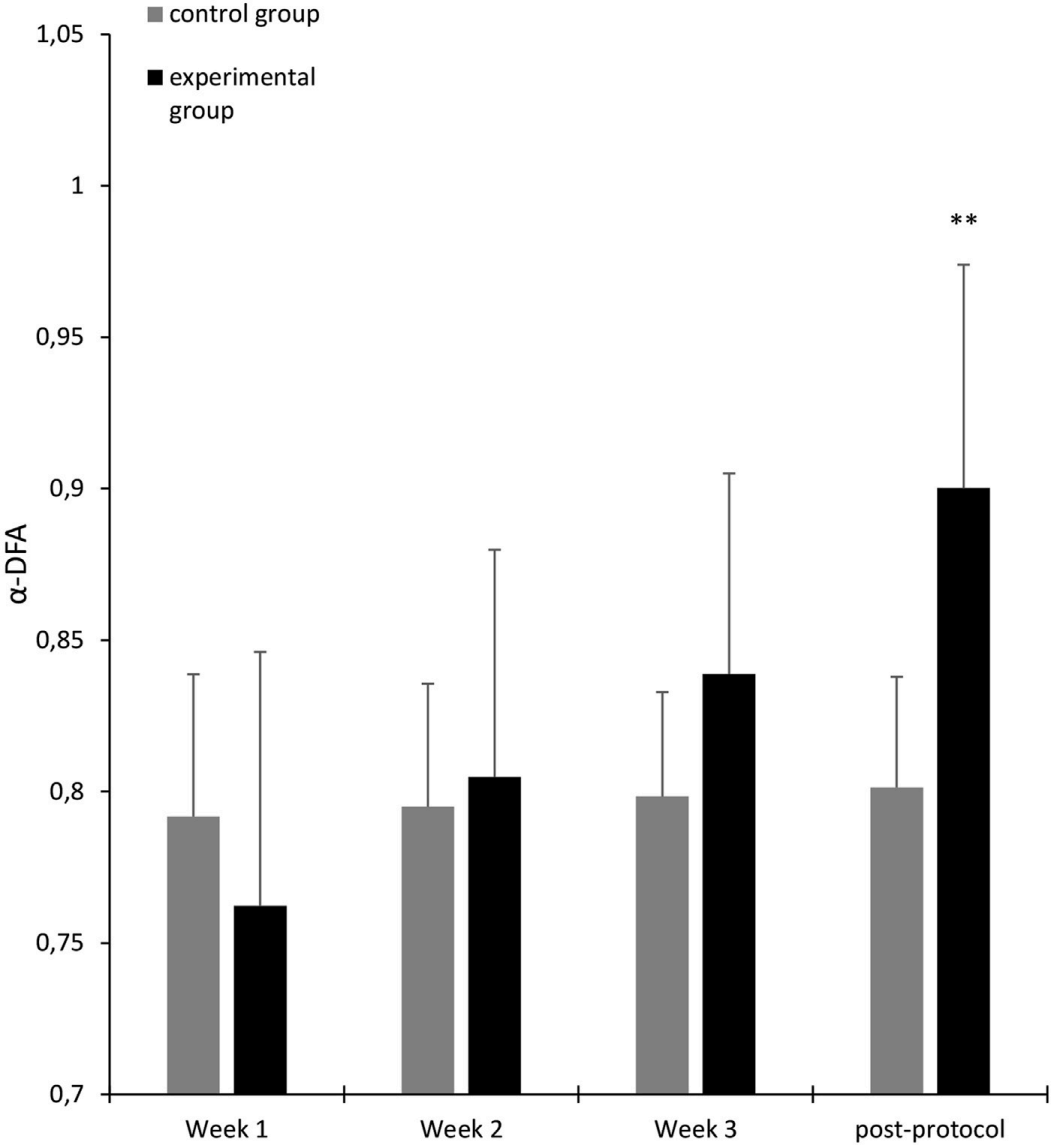
Average α-DFA exponents calculated for participants in solo sequences (black: experimental group, gray: control group), over 3 weeks of trainingand post-protocol test. Error bars represent standard deviation. The significance stars indicate differences compared to Week 1. ∗∗*p* < 0.01.

The results evidenced an increase of walking complexity in the experimental group during the post-protocol test. We report in Table 1 the results of the first 3-way ANOVA performed on the α-DFA exponents calculated during the solo sequences. These results are illustrated in Figure 4. The ANOVA test showed a significant interaction effect between Group and Tests [*F* (3.99) = 13.581, *p* = 0.0000, partial *η* ^2^ = 0.292], and the Fisher LSD post-hoc test indicated that the values obtained in the experimental group during the post-protocol test were higher than those observed during the first week (*p* = 0.000000).

**TABLE 1:** 3-factor ANOVA results: 2 (group) X 4 (test) X 2 (guide).

	F	P	η^2^
Group	3.266	0.079	0.090
Guide	0.527	0.473	0.016
Group X guide	0.852	0.363	0.025
Test	17.229	0.000	0.353
Test X group	13.581	0.000	0.292
Test X guide	0.577	0.631	0.017
Test X group X guide	1.720	0.168	0.049

**FIGURE 5:**
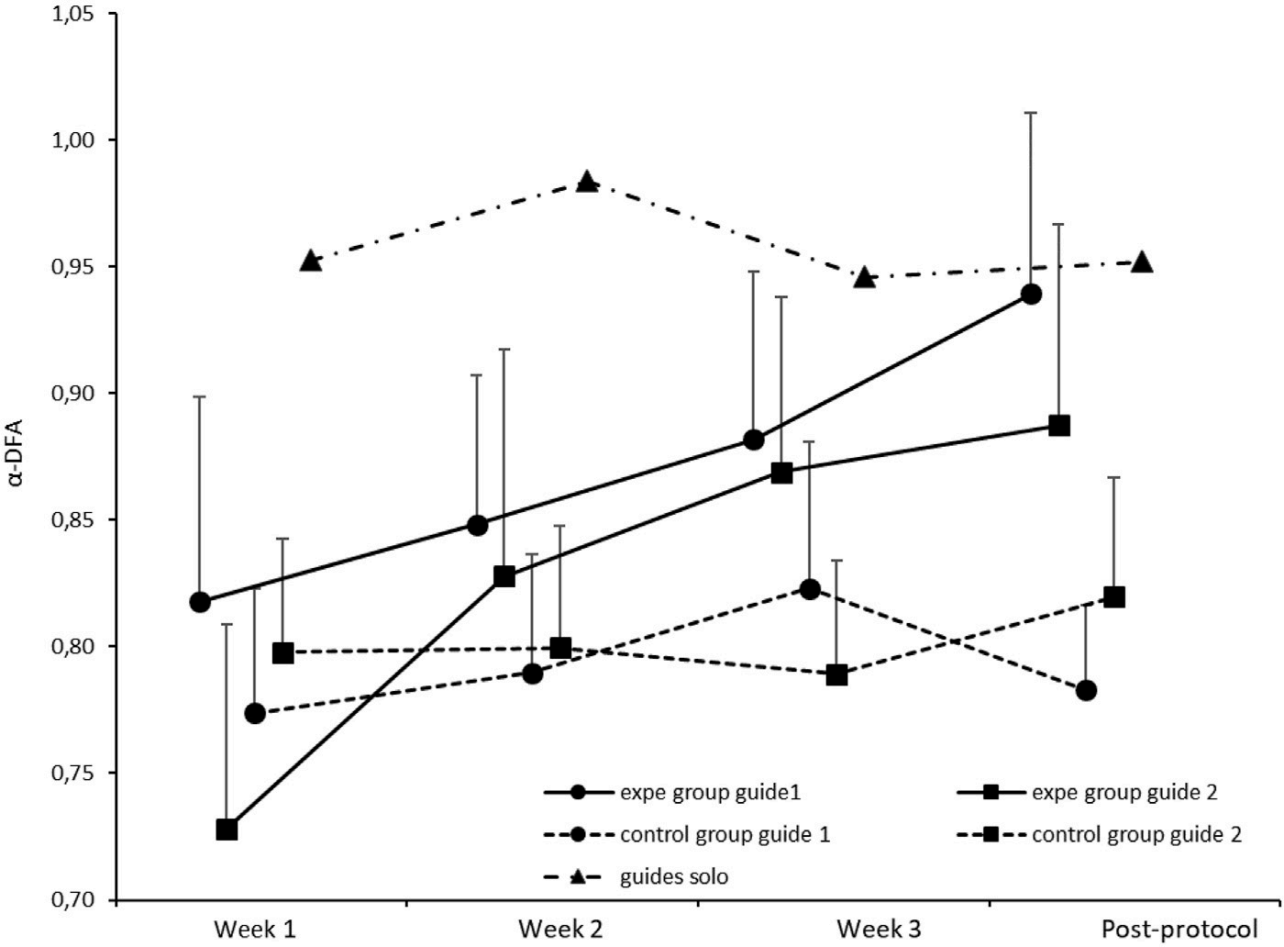
Average α-DFA exponents calculated for participants in solo sequences (solid lines: experimental group, broken lines: control group), over the 3 weeks of training and the post-protocol test. Round markers represent participants supported by Guide 1 (female) and square markers represent participants supported by Guide 2 (male). Error bars represent standard deviation. We also reported the mean α-DFA exponents obtained for the two guides (black triangles).

We noted also that the complexity restoration is not guide-dependent. As mentioned in Table 1, the 3-way ANOVA (week x group x guide) shows no significant difference for the Guide factor on the evolution of mean α-DFA exponents. These results are illustrated in Figure 5 in which we show the evolution of the mean α-DFA exponents over the 3 weeks of the protocol (solo sequences) and the post-protocol test. The continuous lines represent the participants of the experimental group and the broken lines represent the control group. Note that the experimental group had to perform the walk accompanied and synchronized with guide 1 or 2, which was not the case for the control group. Concerning the experimental group, the circle markers represent participants who walked with guide 1 (young woman) and the square markers represent participants who walked with guide 2 (young man). We also reported in this figure the mean α-DFA exponents obtained for the two guides (black triangles).

**FIGURE 6:**
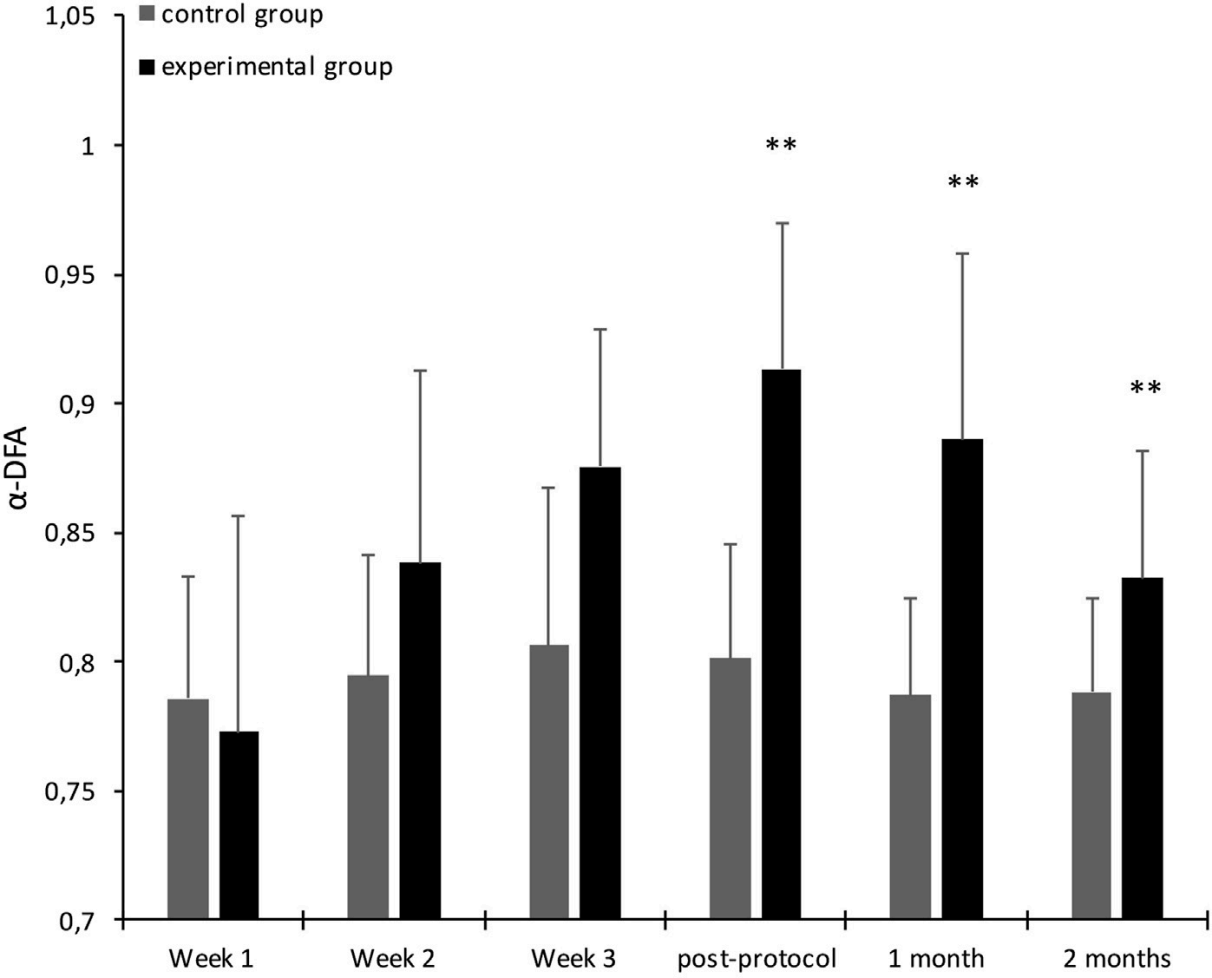
Average α-DFA exponents calculated for participants in solo sequences (black: experimental group, gray: control group), over the 3 weeks of training, the post-protocol test and post-tests. Error bars represent standard deviation. The significance stars indicate differences compared to Week 1. ∗∗*p* < 0.01.

The results of the second ANOVA showed a significant interaction effect between Group and Test [*F* (5.100) = 5.604, *p* = 0.0001, partial *η* ^2^ = 0.219], and the Fisher LSD post-hoc test indicated that there was a significant difference between the measurement at test 1 and the post-test at 1 month (*p* < 0.000) in the experimental group. Additionally, there is no significant difference between the post-protocol test and the post-test at 1 month. These results are illustrated in Figure 6. The post-test at 2 months was also significantly higher than the measurement at week 1 (*p* < 0.00). We report in Table 2 the results of the second 3-way ANOVA performed on the α-DFA exponents calculated during the solo sequences.

**TABLE 2:** 2-factor ANOVA results: 2 (group) X 6 (test).

	F	P	η^2^
Group	11.243	0.003	0.360
Test	8.372	0.000	0.295
Test X group	5.604	0.000	0.219

We present in Tables 3 and 4 the descriptive statistics for the α-DFA values recorded during the solo and duo walking sessions. In their meta-analysis, Ravi et al. (2020) provide evidence that a mean scaling exponent of 0.86 is able to optimally discriminate between young and old participants. Our results (see Table 3) show that during the solo trial performed at the beginning of the experiment, the mean *α* exponent was in both groups below this threshold (exp. group, *α* = 0.76, *SD* = 0.08; control group, *α* = 0.79, *SD* = 0.05). During the post-protocol solo trial, the control group remained below this threshold (*α* = 0.80, *SD* = 0.04), while the exp. group was above (*α* = 0.90, *SD* = 0.07), which is consistent with our hypothesis of a restoration of complexity in the experimental group. The results are similar for the solo trials performed 1 month after the training protocol (exp. group, *α* = 0.89, *SD* = 0.07; control group, *α* = 0.79, *SD* = 0.03). During the 2-month post-test, however, the mean exponent in the experimental group fell back below the threshold, even though it remained statistically higher than the initial value (*α* = 0.83, *SD* = 0.05).

**TABLE 3:** Descriptive statistics of α-DFA data in solo sessions.

	Week 1	Week 2	Week 3	Post-protocol	1 month	2 month
Mean.Exp.group	0.76	0.80	0.84	0.90	0.89	0.83
SD.Exp.group	0.08	0.07	0.07	0.07	0.07	0.05
IQR.Exp.group	0.11	0.09	0.09	0.09	0.11	0.08
Sizes.Exp.group	20	20	20	20	15	12
Mean.Contr.group	0.79	0.79	0.80	0.80	0.79	0.79
SD.Contr.group	0.05	0.04	0.03	0.04	0.03	0.04
IQR.Contr.group	0.08	0.06	0.05	0.06	0.02	0.05
Sizes.Contr.group	16	16	16	16	121	10

Mean.Exp.group, mean of α-DFA in the experimental group; SD.Exp.group, Standard deviation of α-DFA in the experimental group; IQR.Exp.group, interquartile ranges of α-DFA in the experimental group; Sizes, sample sizes of the experimental group; Mean.Contr.group, mean of α-DFA in the control group; SD.Contr.group, Standard deviation of α-DFA in the control group; IQR.Contr.group, interquartile ranges of α-DFA in the control group; Sizes, sample sizes of the control group.

**TABLE 4:** Descriptive statistics of α-DFA data in duo sessions.

	Week 1	Week 2	Week 3
Mean.Exp.group.participants	0.85	0.86	0.83
SD.Exp.group.participants	0.08	0.09	0.09
IQR.Exp.group.participants	0.10	0.15	0.12
Sizes.Exp.group.participants	20	20	20
Mean.Exp.group.guides	0.86	0.88	0.84
SD.Exp.group.guides	0.08	0.09	0.10
IQR.Exp.group.guides	0.13	0.09	0.10
Sizes.Exp.group.guides	20	20	20

Mean.Exp.group.participants, mean of α-DFA of participants in the experimental group; SD.Exp.group.participants, Standard deviation of α-DFA of participants in the experimental group; IQR.Exp.group.participants, interquartile ranges of α-DFA of participants in the experimental group; Sizes, sample sizes of the duo sessions in the experimental group; Mean.Exp.group.guides, mean of α-DFA of the guides in the experimental group; SD.Exp.group.guides, Standard deviation of α-DFA of guides in the control group; IQR.Exp.group, interquartile ranges of α-DFA of guides in the experimental group; Sizes, sample sizes of the duo sessions in the experimental group.

### 3.2 Clinical impact of the experimental protocol

**FIGURE 7:**
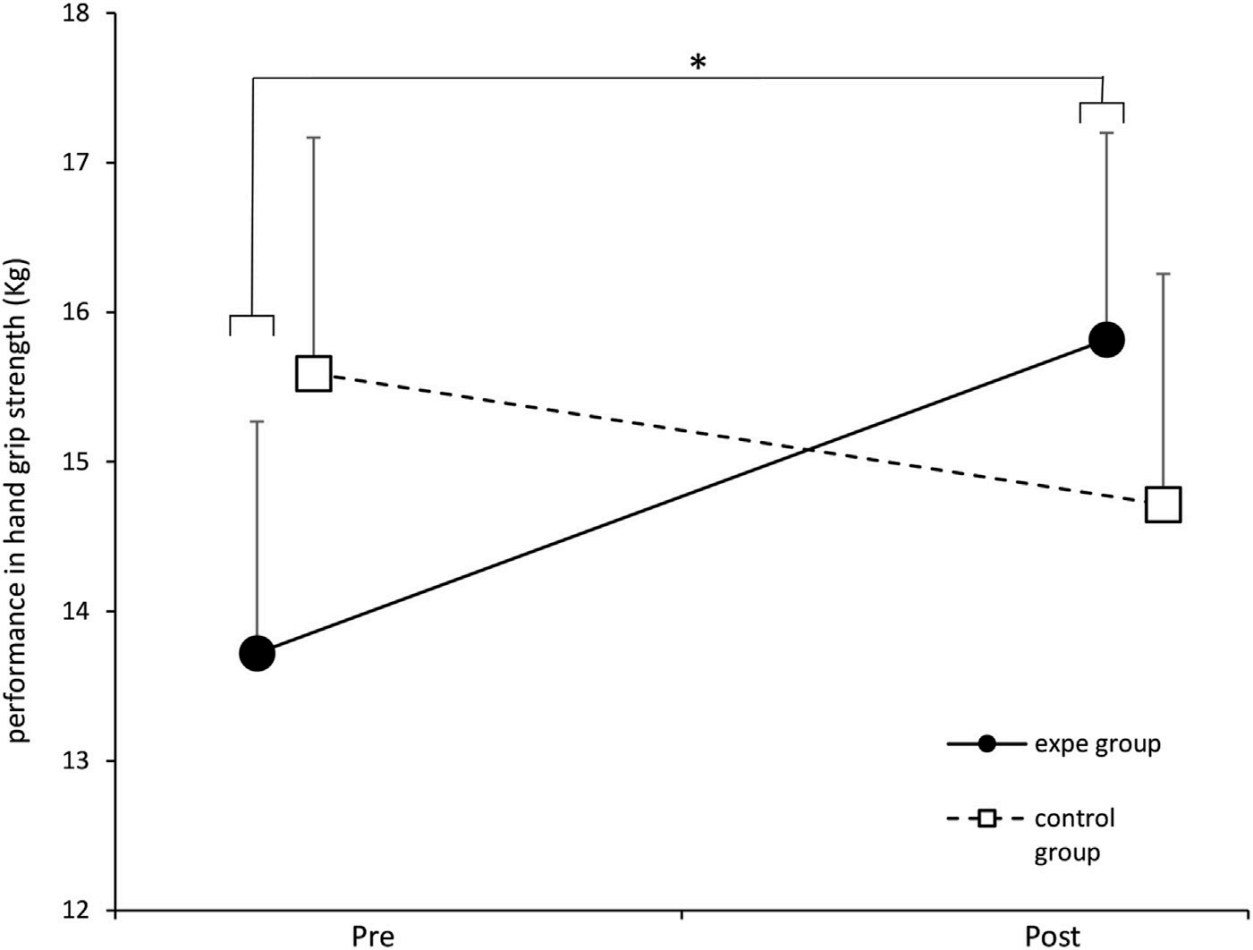
Evolution of the average performance in hand grip strength (addition of the 2 hands) of the experimental group and the control group before and after the training protocol. ∗ p < 0.05. Error bars represent standard deviation.

In Figure 7, we report the evolution of the performance in hand grip strength for the experimental group and the control group before and after participation in the experimental walking protocol. The solid line represents the mean of the results of the experimental group and the broken line represents the mean of the results of the control group.

The two-factor ANOVA shows a significant effect of the protocol on the performance in hand grip strength [*F* (1.34) = 4.824, *p* = 0.03497, partial *η* ^2^ = 0.124]. The Fisher LSD post-hoc test revealed a significant improvement in hand grip strength performance in the experimental group after their participation in the walking protocol (*p* = 0.0178). Regarding the control group, we did not note any significant effect of the protocol on the performance in hand grip strength.

**FIGURE 8:**
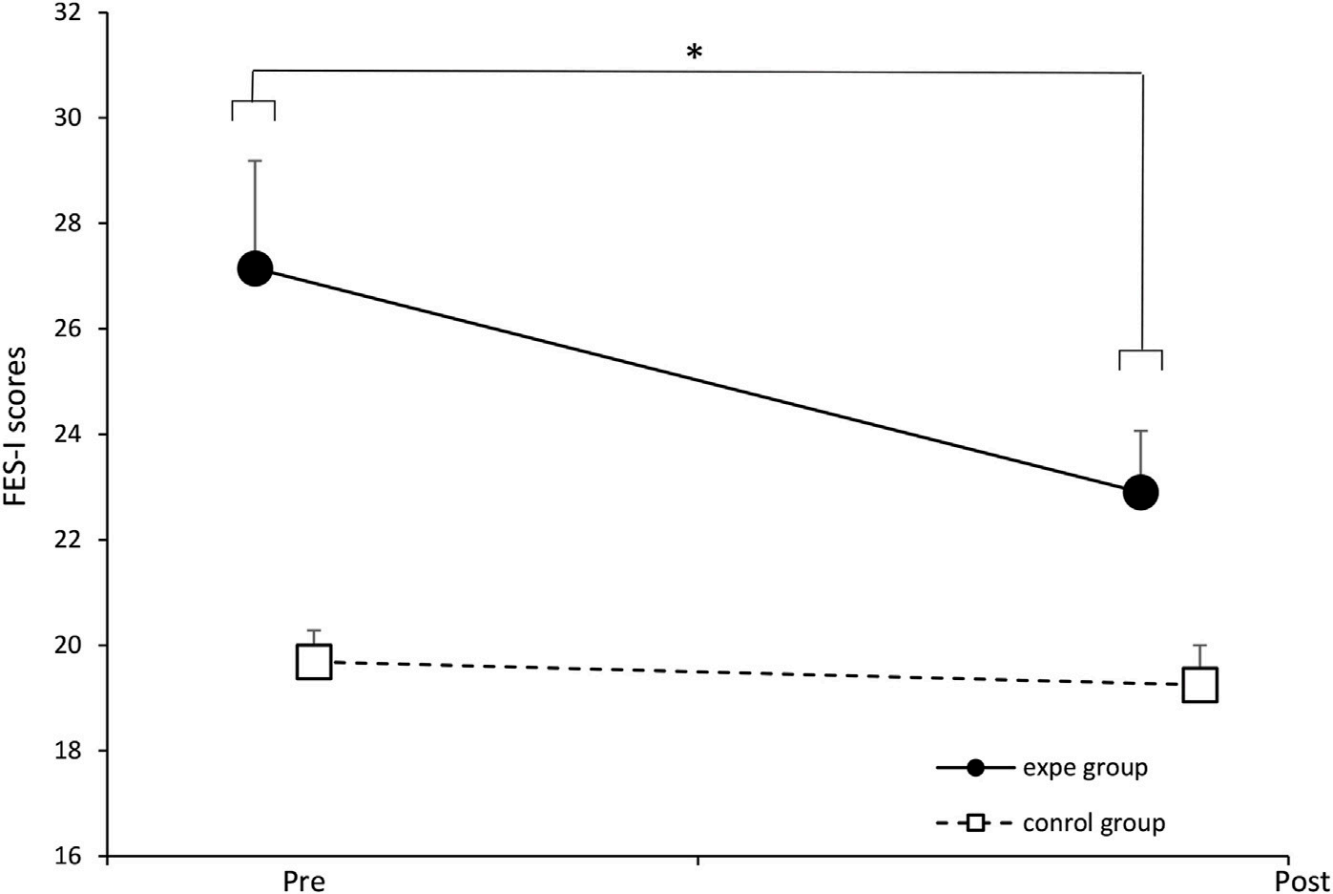
Evolution of the mean scores of the experimental group and the control group before and after the training protocol. ∗ *p* < 0.05. Error bars represent standard deviation.

We report in Figure 8 the evolution of the score of the FES-I questionnaire before and after the 3 weeks of training. The solid line represents the experimental group and the broken line represents the control group. We applied a two-way ANOVA which showed a significant effect of the protocol on the FES-I score [*F* (1.34) = 4.25, *p* = 0.04697, partial *η* ^2^ = 0.1111]. The Fisher LSD post-hoc test revealed a significant improvement in the FES-I score in the experimental group before and after their participation in the walking protocol, *p* = 0.001. This result shows that the experimental protocol has an effect on apprehension upon falling. The participants of the experimental group presented scores revealing a fear of falling significantly lower compared to the scores recorded before their participation in our experimental protocol. This improvement in the FES-I score only concerns the experimental group.

**FIGURE 9:**
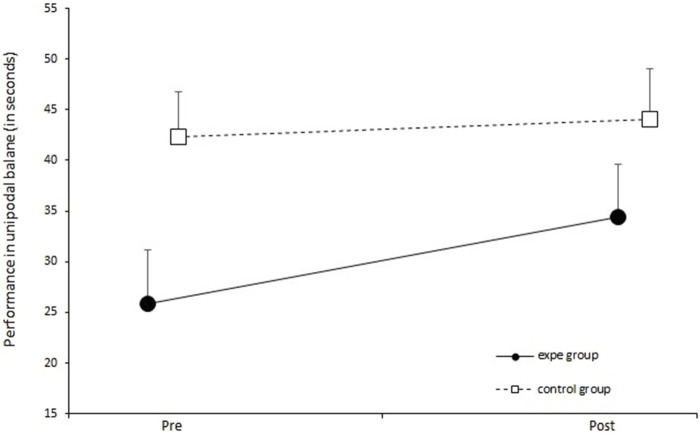
Evolution of performance (sum of the 2 legs) in unipodal balance before and after the training protocol for the control group and the experimental group. Error bars represent standard deviation.

In Figure 9, we report the evolution of the performance of participants in unipodal balance before and after their participation in the walking protocol. The solid line represents the average performance of the experimental group and the broken line represents the average performance of the control group. The two-factor ANOVA does not show any significant effect of the protocol on performance in unipodal balance [*F* (1.34) = 1.5548, *p* = 0.22199, partial *η* ^2^ = 0.044]. The pairing of complexities had no effect on improving the unipodal balance in our participants.

**FIGURE 10:**
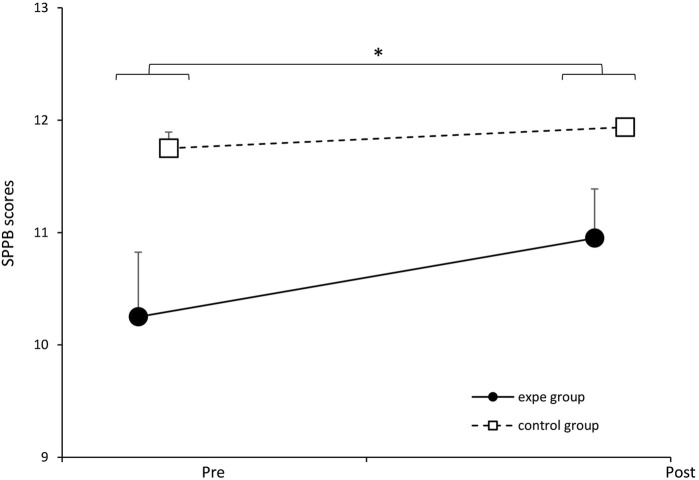
Evolution of the SPPB score of the experimental group and the control group before and after the training protocol. ∗p < 0.05. Error bars represent standard deviation.

We illustrate in Figure 10 the evolution of the SPPB score of the participants before and after their participation in our experimental protocol. The solid line represents the average performance of the experimental group and the dotted line represents the average performance of the control group. The two-way ANOVA does not show a significant effect of the interaction between the two factors (group/week) [*F* (1.34) = 3.222, *p* = 0.081, partial *η* ^2^ = 0.087]. Nevertheless, ANOVA reveals a significant effect of the protocol on performance at the SPPB regardless of the group of participants [*F* (1.34) = 9.662, *p* = 0.003, partial *η* ^2^ = 0.2213].

Therefore, our results show that gait training could have a positive effect in the SPPB score (indicating better functional status of the participants). This reflects a lack of association between the pairing of complexities induced in the experimental group and the improvement in the score of the SPPB test.

**FIGURE 11:**
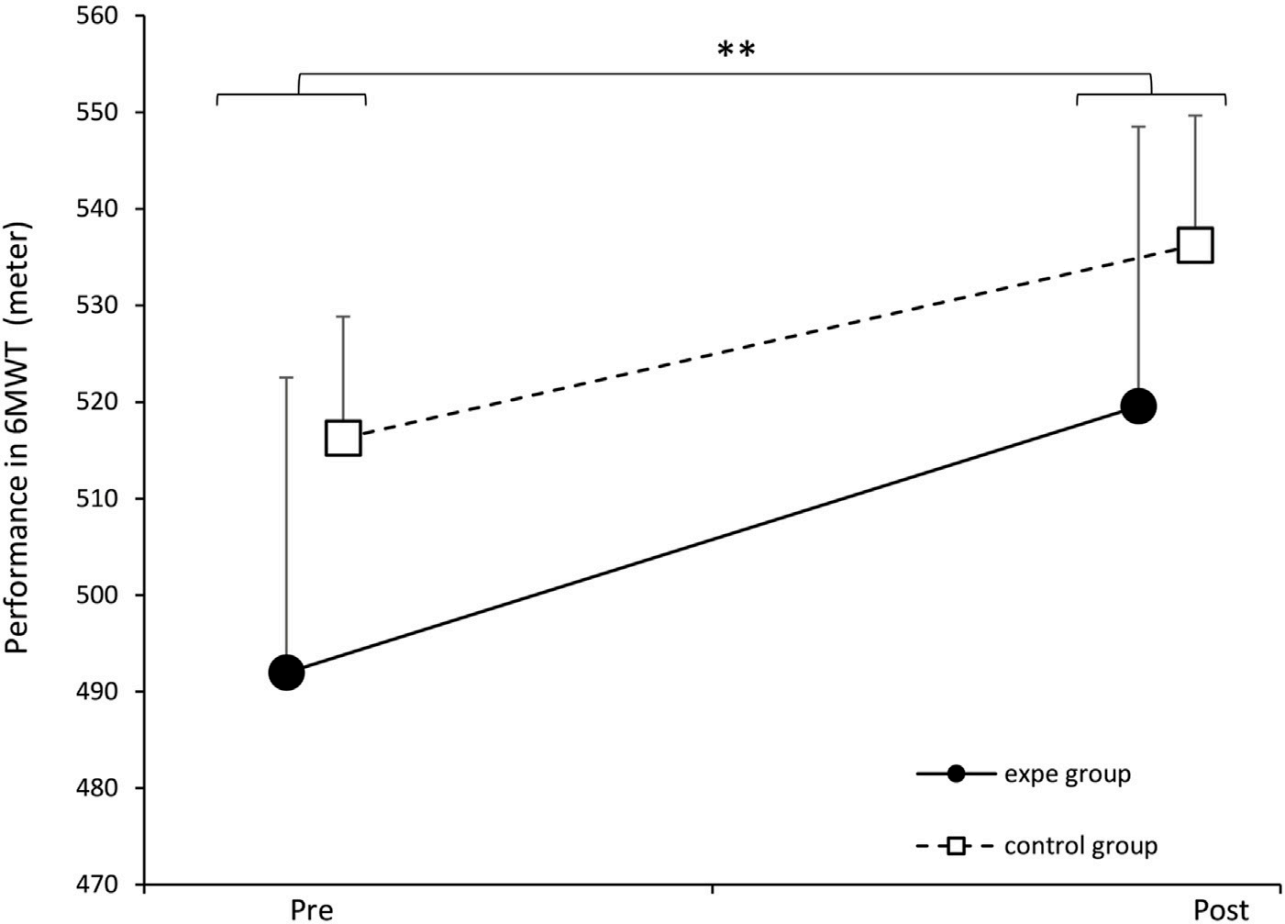
Evolution of the average performance at 6MWT of the experimental group and the control group before and after the training protocol. Error bars represent standard deviation. *p* < 0.01. Error bars represent standard deviation.

We report in Figure 11 the evolution of the participants’ performance on the 6-min walk test (6MWT) before and after the 3 weeks of training. The solid line represents the experimental group and the broken line represents the control group. We applied a two-way ANOVA which did not show a significant effect of the interaction between the two factors (group/week) [*F* (1.34) = 0.689, *p* = 0.41234, partial *η* ^2^ = 0.0199]. However, we noted a significant effect of the training protocol on 6MWT performance in all participants (experimental and control groups) [*F* (1.34) = 27.022, *p* = 0.00000, partial *η* ^2^ = 0.4428]. Consequently, this test cannot explain the effect of restoring complexity observed in the experimental group, but it simply makes it possible to show an improvement in endurance in all the participants thanks to training.

**FIGURE 12:**
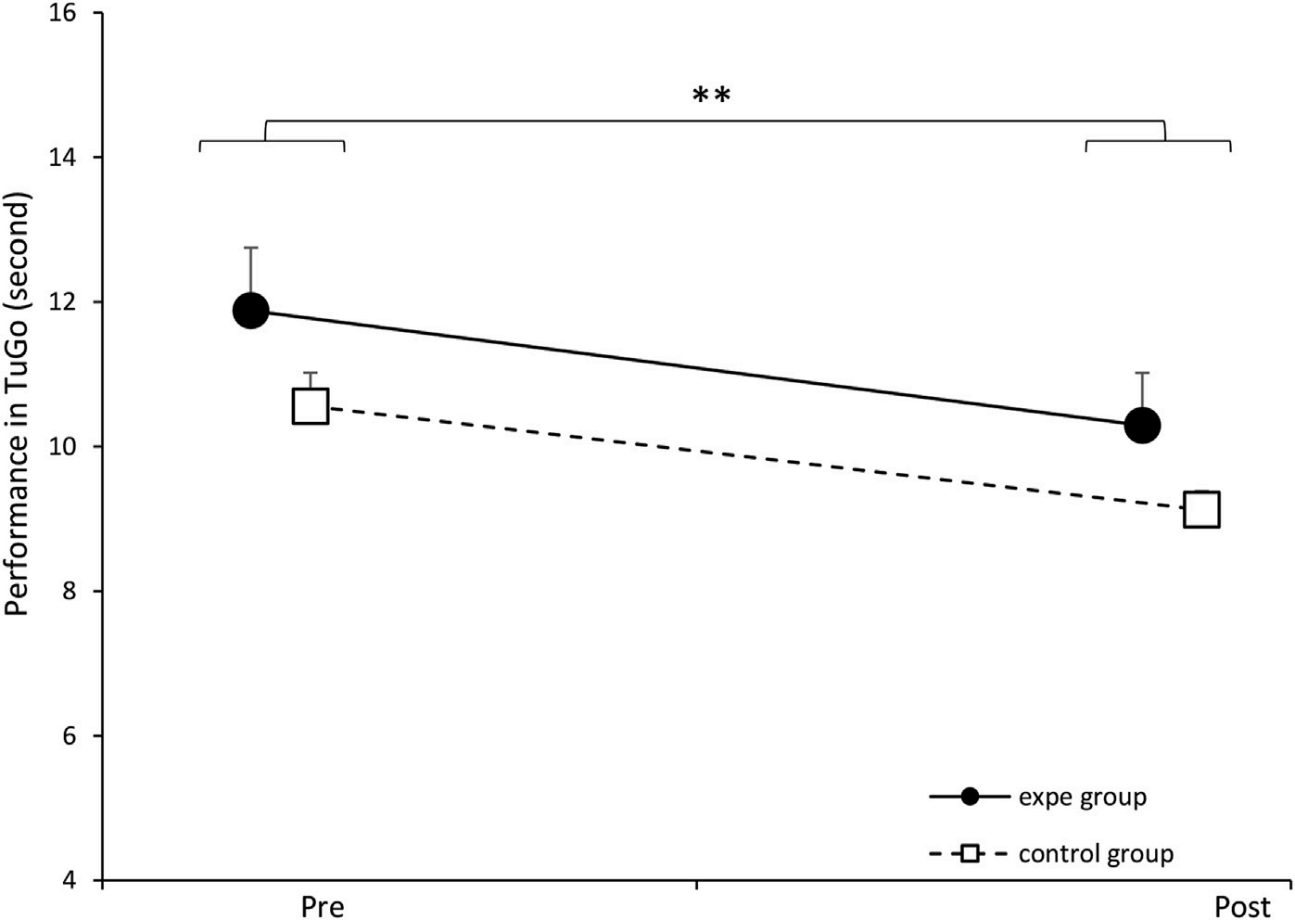
Evolution of the average TuGo performance of the experimental group and the control group before and after the training protocol. ∗*p* < 0.01. Error bars represent standard deviation.

In Figure 12, we illustrate the evolution of the participants’ performance on the TuGo test before and after their participation in the experimental protocol. The solid line represents the experimental group and the broken line represents the control group. two-way ANOVA did not show a significant effect of the interaction between the two factors (group/week) [*F* (1.34) = 0.056, *p* = 0.81372, partial *η* ^2^ = 0.0017]. However, we noted a significant effect of the training protocol on TuGo performance in all participants (experimental and control groups) [*F* (1.34) = 20.696, *p* = 0.00006, partial *η* ^2^ = 0.3784]. These results show that prolonged walking training improves performance on the TuGo, but this test did not discriminate performance between the two groups.

## 4 Discussion

All our hypothesis around the restoration of complexity have been validated: When we invite an older person to walk in synchrony, arm-in-arm, with a young, healthy partner, the synchronization is mainly dominated by a complexity matching effect. This hypothesis was verified through the WDCC analysis, which revealed a positive peak at lag 0 showing immediate synchronization between the interacting systems (participants and guide). In addition, this convergence of complexities appears from the first duo sequences performed during the training protocol. These results confirm our previous results (Ezzina et al., 2021) and those of Almurad et al. (2018).

Moreover, our results allowed once again to validate the fundamental results of Mahmoodi et al. (2018), showing that when two systems of different levels of complexity interact, the more complex tends to attract the less complex.

This study also confirmed that the prolonged experience of complexity matching (during a 3-week protocol) allows the restoration of the complexity of the locomotion system in older people, as evidenced by the increase α-DFA exponents at the end of the protocol and during post-tests. Therefore, this experiment, like our previous work (Ezzina et al., 2021) and the work of Almurad et al. (2018) show that 3 weeks of intensive synchronized walking practice can provide a significant restoration of complexity. Moreover, the absence of a significant evolution of the α-DFA exponents in the control group indicates that the effect of the restoration of the complexity depends only on the complexity matching effect. Therefore, physical activity is not sufficient to induce an improvement in complexity in stride dynamics (the control group achieved the same training load as the experimental group).

We obtained similar results with both guides, suggesting that the conclusions of our previous work (Ezzina et al., 2021) and those of Almurad et al. (2018) could be generalized. Indeed, during the two previous experiments using this kind of protocol, the guides were always doctoral students (the experimentation being part of their doctoral work), which could represent an experimental bias (Gidron et al., 2020; Doyen et al., 2012). In the present study, our guides were on a civic service mission within our research unit for 6 months, and they had no direct link with the scientific project. Therefore, our results showed that the complexity restoration effect obtained in the experimental group was not dependent on the guide. This represents an essential result, making it possible to pursue with greater confidence the prospects for rehabilitation and prevention offered by this type of protocol.

Finally, this work revealed a persistence of the complexity restoration effect up to 2 months post-protocol. Nevertheless, we observe a trend towards degradation during the 2-month post-test, which leads us to consider new forms of reminder protocols in order to avoid a total degradation of the effect obtained.

During this exploratory study, we wanted to set up a battery of clinical tests conventionally used and widespread in the literature and in geriatrics departments for measuring the risk of falling and/or frailty in the elderly. This tests battery should not exceed 30 min (including breaks) in order to avoid the effects of fatigue.

It was expected to record performance improvements in all the participants of the experiment (control group and experimental group). This improvement would be due to the regular walking training induced by our experimental protocol (Okubo et al., 2016; Shimada et al., 2003). However, we hypothesized to obtain significantly greater improvements in the experimental group compared to the control group. Indeed, we obtained an improvement in performance of all participants (two groups) on three clinical tests: TuGo, 6MWT and SPPB. Nevertheless, only two clinical tests allowed to distinguish between the two groups: the hand grip strength test and the FES-I questionnaire. Note that the hand grip strength test, unlike the rest of the physical tests carried out, is the only test carried out with an automated electronic device (electronic dynamometer), and could be more objective and reliable, as compared to the other tests of our battery.

Therefore, given that hand grip strength is indicative of overall health status and mortality risk, the results of our study support a possible link between the restoration of complexity recorded in the experimental group and the improvement in the overall health, and a reduction in the risk of mortality. This result is very original and promising, but we are aware that an increase in the sample of participants is necessary to confirm it. Furthermore, we emphasize that the effect size is medium since we obtained a η^2^ = 0.124 (threshold for large effect size = 0.14), so an increase in the sample of participants would be interesting to confirm this result.

The FES-I was the only questionnaire in our test battery. It allows to assess fear of falling. The results allow to establish a link between the restoration of the complexity of the locomotion system and the reduction in apprehension of falling. We obtained a medium sized effect (*η*2 = 0.1111), so an increase in the sample of participants seems also necessary to confirm this result. More, Delbaere et al. (2010) proposed a cut-off point set at 23, above which an individual is considered to have a high fear of falling. In the experimental group, the pre-test average was 27.15 (*SD* = 9.10), which decreased to 22.9 (*SD* = 5.23) after the protocol. This result is promising, as it suggests that by restoring complexity, we were able to reduce this fall risk factor below the cut-off point.

Note that the health context linked to COVID-19 led us to interrupt our experiments and lose experimental data.

It is also important to emphasize that detecting significant changes in this questionnaire within a 3-week interval is likely challenging. In future studies, it would be interesting to re-assess participants at post-tests (e.g., at one and 2 months) to potentially observe major changes in their fear of falling.

Regarding the SPPB test, we note that it may not be suitable for an independent elderly population due to its lack of discrimination. Our pre-test results showed scores that were already close to the maximum, limiting the ability to observe changes in the post-tests.

Our results on the Timed Up and Go (TuGo) test suggest that gait training helps reduce frailty in older adults. According to Shumway-Cook et al. (2000), a time of 11 s or more is considered an indicator of frailty. In our experimental group, the average pre-test score was 11.88 s (*SD* = 3.89), which improved to 10.29 s (*SD* = 3.25) in the post-test. However, the TuGo test did not allow to distinguish between the control and experimental groups, possibly due to the limited sample size or a lack of sensitivity in the test.

We also note a significant improvement in 6MWT performance in both groups. However, this test may not be suitable for assessing the restoration of gait complexity. Given that our participants were independent seniors, their pre-test performances were already relatively high (Hanifah et al., 2019; Bautmans et al., 2004), which may have limited the ability to detect differences between the two groups. Thus, while our protocol positively impacted the overall health status of the participants (Bautmans et al., 2004), the 6MWT may not be sensitive enough to distinguish between solo walking and duo walking (complexity matching effect). More, the 6MWT is the test that most closely resembles the type of activity performed during the protocol (walking) and is considered an indicator of overall health status. Therefore, it would likely be beneficial to increase the sample size to better differentiate between the two groups.

Our results indicate that the protocol had no effect on unipodal balance. Although balance is directly linked to fall risk (Sakamoto et al., 2006), 3 weeks of gait training did not improve this factor. We emphasize that complexity refers to a holistic concept, which may not be adequately captured by a test assessing a single function in isolation.

We believe that our clinical tests, measured manually with a stopwatch and assessing a specific function, cannot objectively capture the effect of locomotion complexity restoration. This is because complexity is a global and non-specific concept, and these tests are likely not precise or sensitive enough.

This study is the first to attempt to reconcile the fundamental question of restoring the complexity of a deficient system and the hypothesis of Hausdorff et al. (1997) suggesting a link between the loss of complexity and the propensity to fall. Nevertheless, the protocol that we propose remains difficult for the participants (approximately 36 km of walking during the 3 weeks of the protocol). This obliged us to recruit autonomous participants who have no contraindications to the practice of regular physical activity. This could also explain a moderate loss of complexity in our participants. Of course, it would be interesting to adapt this protocol for more fragile patients (parkinsonian patients, post-stroke, etc.). In addition, a battery of manually measured tests could be insufficient to translate a complexity restoration effect, therefore, additional efforts are necessary to imagine reliable and reproducible objective tests (examples: biological tests) to try to translate the link between restoration of complexity and reduced risk of falls in older adults.

The main result of this experiment is the confirmation that it is possible to restore the complexity of the locomotor system in older people. This restoration is expressed during tests carried out alone and is sustainable, at least 2 months after the administration of the treatment. The restoration of complexity must be understood as an enrichment of connectivity within a physiological network, an increase in the interactions between its multiple components. Unlike classic approaches that often target their interventions on one of the elements of the system, the aim here is to work at the level of the network itself, conceived as dominated by the dynamics of the interactions between its components (Balagué et al., 2020; West, 2024; West and Mudaliar, 2025).

## Data Availability

The original contributions presented in the study are included in the article/supplementary material, further inquiries can be directed to the corresponding author.
